# Supplementary material for: Gaze-behaviors of runners in a natural, urban running environment
Source: PLoS One. 2020 May 19;15(5):e0233158. doi: 10.1371/journal.pone.0233158 (PMC7237013; doi:10.1371/journal.pone.0233158)
Supplement: Attachment — Submitted filename: Response to Reviewers_MC.docx [file pone.0233158.s004.docx]

We want to thank the editor, Dr. Liu, and our anonymous editor for their comments. We were able to accommodate your suggestions and we think that the changes have improved the manuscript. We are grateful for the opportunity to resubmit our manuscript for publication.

Response to Reviewers

Reviewer #1: The revision of the manuscript reasonably address all the comments with one exception. Regarding the switch from using Pearson correlation to Spearman rank correlation, corresponding changes/edits shall be made at the section of ‘Data analysis’ (lines 339-344) and the figure illustrating the correlation between fixation number and running speed shall be updated to reflect the tested (i.e., rank) instead of original variable metrics.

I would endorse the acceptance of this manuscript after seeing these changes, which is necessary for a coherently presented manuscript.

***Thank you for your comment, we are sorry we did not address this point fully in our last revision. We have adjusted line 339 to state “Spearman’s rank correlation” rather than “ordinary least squares regression.” We have also removed the regression line from the Spearman’s rank correlation in Figure 6.***
